# Maternal childbirth experience and induction of labour in each mode of delivery: a retrospective seven-year cohort study of 95,051 parturients in Finland

**DOI:** 10.1186/s12884-022-04830-9

**Published:** 2022-06-23

**Authors:** Johanna M. Joensuu, Hannu Saarijärvi, Hanna Rouhe, Mika Gissler, Veli-Matti Ulander, Seppo Heinonen, Paulus Torkki, Tomi S. Mikkola

**Affiliations:** 1 Helsinki University Hospital, Obstetrics and Gynecology, Haartmaninkatu 2 PL 140, Helsinki, 00029 Finland; 2 Present Address: Department of Public Health, University of Helsinki, Helsinki, Finland; 3 Tampere University, Faculty of Management and Business, Tampere, Finland; 4 University of Helsinki and Helsinki University Hospital, Obstetrics and Gynecology, Helsinki, Finland; 5 Finnish Institute for Health and Welfare, Information Services Department, Helsinki, Finland; 6 Karolinska Institute, Department of Molecular Medicine and Surgery, Stockholm, Sweden; 7 Academic Primary Health Care Centre, Stockholm, Region Stockholm Sweden; 8 Folkhälsan Research Center, Biomedicum, Helsinki, Finland

**Keywords:** Maternal experience, Giving birth, Pregnancy, Maternity, Induced labour, Delivery method

## Abstract

**Background:**

Childbirth experience has been shown to depend on the mode of delivery. However, it is unclear how labour induction influences the childbirth experience in different modes of delivery. Thus, we assessed the childbirth experience among mothers with spontaneous and induced labours.

**Design:**

A retrospective cohort study.

**Setting:**

Childbirths in four delivery hospitals in Helsinki and Uusimaa District, Finland, in 2012-2018.

**Sample:**

95051 childbirths excluding elective caesarean sections.

**Methods:**

Obstetric data combined to maternal childbirth experience measured by Visual Analogue Scale (VAS) was analysed with univariate linear modelling and group comparisons. The primiparas and multiparas were analysed separately throughout the study due to the different levels of VAS.

**Main outcome measures:**

Maternal childbirth experience measured by VAS.

**Results:**

The negative effect of labour induction on the childbirth experience was discovered in each mode of delivery. Operative deliveries were perceived more negatively when they were preceded by labour induction. The rate of poor childbirth experience (VAS≤5) was higher for mothers with labour induction (ORs varying from 1.43 to 1.77) except in emergency caesarean sections. The negative effect of labour induction was smaller than the effect of mode of delivery, while successful vaginal delivery with induction (mean_PRIMI_=8.00 [95% CI 7.96–8.04], mean_MULTI_=8.50 [8.47–8.53]) was perceived more positive than operational deliveries with spontaneous labour (means_PRIMI_≤7.66 [7.61–7.70], means_MULTI_≤7.96 [7.89–8.03]). However, labour induction more than doubled the risk of caesarean section for both primiparas and multiparas.

**Conclusions:**

Labour induction generates more negative experiences for both primiparas and multiparas. The negative effect of labour induction is detected for all modes of delivery, being worst among labour induction resulting in operative delivery. The parturients facing cumulative obstetric interventions require special support and counselling during and after delivery.

**Supplementary Information:**

The online version contains supplementary material available at 10.1186/s12884-022-04830-9.

## Introduction

Childbirth as a physical, social and emotional event has essential immediate and long-term implications on the health and wellbeing of a mother and the entire family [1]. It is also shown that poor or traumatic childbirth experiences have an impact on future reproduction [2–4]. The visual analogue scale (VAS) as a simple measure for childbirth experience may not fully capture the holistic nature of the childbirth phenomenon, but its validity and reliability as an overall satisfaction measure has been comprehensively demonstrated [5–8].

During the past decade several meta-analyses and reviews have indicated benefits benefits from labour induction for the neonate without increased risk for the mother in term and post-term gestations [9, 10]. These studies indicated more desirable perinatal outcomes, as induction decreased the numbers of neonate deaths and lowered the need for the neonatal intensive care unit, and decreased the need for caesarean sections as well. However, the data have been criticised on methodological deficiencies due to inappropriate control groups and different timings of compared trials [11–13]. The obstetrical arguments for induction in end term or post-term gestations are undeniable [12]. Nevertheless, the association between induction of labour and the maternal experience of childbirth is poorly understood [8, 14]. It has been shown that successfully induced labour resulting in vaginal delivery did not negatively affect birth outcomes but had a partial negative effect on the childbirth experience [15, 16]. In addition, childbirth experience may also depend on the mode of delivery [2, 16–19].

In this study we investigate how labour induction influences the childbirth experience in different modes of delivery. We distinguish between spontaneous and induced labours and, consequently, address their effects through actual mode of delivery for the childbirth experience.

## Data and methods

The childbirth experience is measured by VAS in the postpartum unit of the delivery hospital. The VAS scale in this study is from ‘very negative childbirth experience’ (1) to ‘very positive childbirth experience’ (10). The VAS was collected as a part of a conversation with a midwife before discharge from the postpartum unit. The midwives have been instructed to pursue as safe atmosphere as possible to avoid biased responses. The women rating their childbirth experience with the score of 5 or less on the VAS scale were offered an opportunity to receive additional support.

The VAS is combined with the Medical Birth Register data using the mother’s identification code, given for all citizens and permanent residents of Finland. The Medical Birth Register is a comprehensive medical register kept by the Finnish Institute for Health and Welfare including antenatal, perinatal and postpartum information from all mothers and infants up to 7 days. The data were pseudonymised and therefore parturients’ identities could not be detected in this data.

**Fig. 1:**
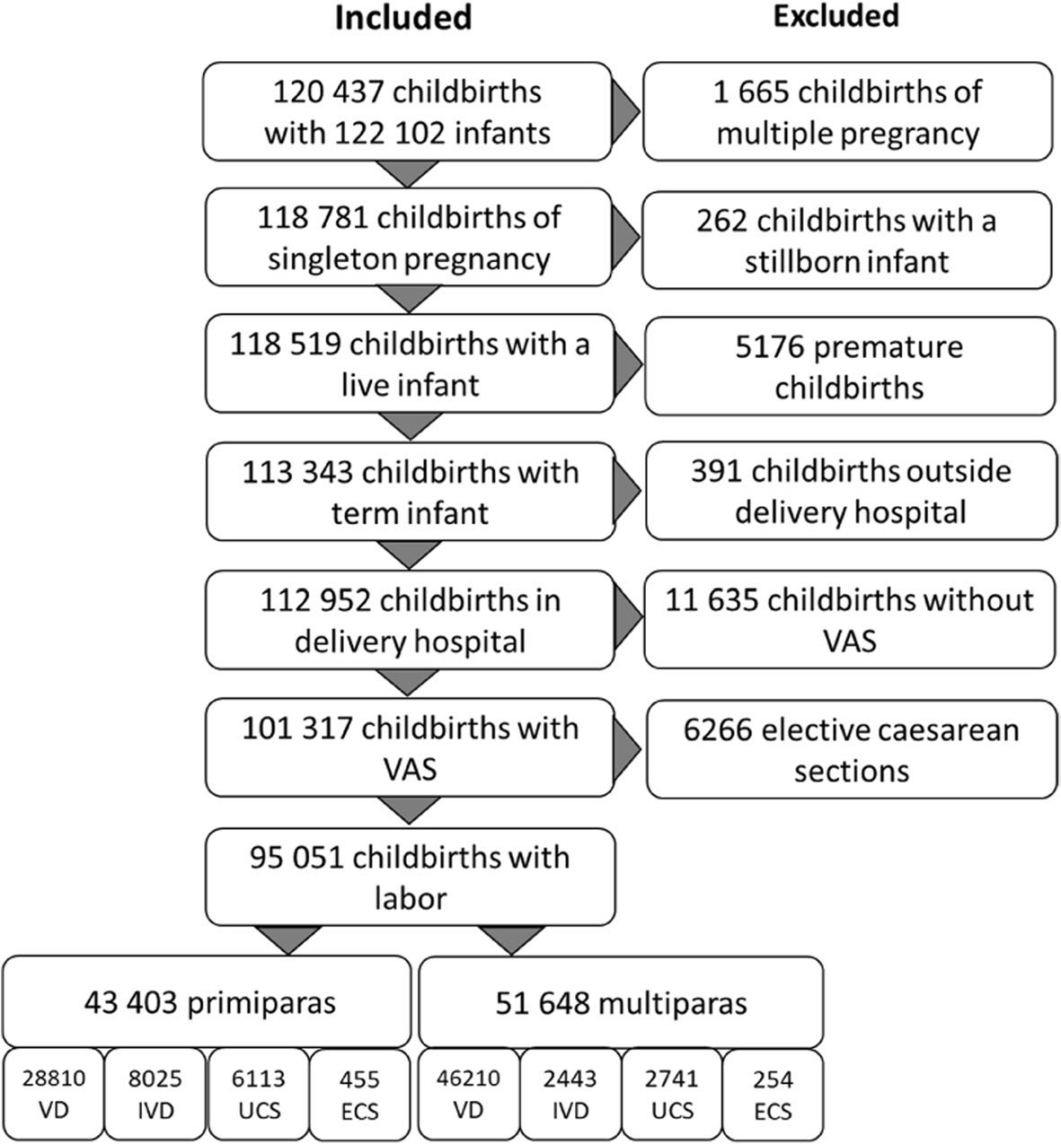
Data inclusion criteria and sample size

This study includes all singleton live births at term (gestational age ≥37) in Helsinki and Uusimaa District hospitals from January 2012 to December 2018. The childbirth experience and labour induction in mode of delivery groups being the key concepts of this study, the parturients without these variables were excluded (*n*=11 635) as well as those having elective caesarean sections (*n*=6 266). The data inclusion criteria are depicted in Fig. 1. There are specific disparities between primiparous and multiparous women when childbirth and mode of delivery are considered [20–22] and therefore, these groups are analysed separately throughout the study. The mode of delivery was classified into four categories: unassisted (VD) and instrumental vaginal deliveries (IVD) as well as urgent (UCS) and emergency caesarean sections (ECS).

The Institutional review board gave the permission (HUS/483/2020) to use the data and waived the requirement of the informed consent for the study since it was a register-based study.

### Analysis

The analyses of this study were conducted in three phases. First, we used two-way analysis of covariance to discover the effects of labour induction and mode of delivery on the childbirth experience. This method assumes the response variable – the childbirth experience by VAS – as continuous and allows controlling for the effects of both binary and continuous confounding variables. Two insights – considering the childbirth experience continuous by nature and ten-point scale being too wide to be analysed in categories - allowed us to utilise analysis of covariance. Assuring the results being consistent between different analysing methods, parallel analyses using generalized linear methods were conducted. Statistical analyses were performed using IBM SPSS Statistics 25 software.

To clarify the association between two factors – labour induction and mode of delivery – and childbirth experience, several confounding variables were included in the second phase of analysis. Confounding variables associated with the childbirth experience were selected based on the previous research, data availability and initial data analysis. Reasonable categories were discovered using appropriate illustrations and statistical tests according to the association between each variable and the childbirth experience. The main goal of this process was to preserve the salient information while compressing it in a few categories to enable unambiguous interpretation. Chi-Square test was used to detect significant differences between spontaneous and induced labour groups Table 1.

**Table 1:** The characteristics of spontaneous and induced labour groups for primiparas and multiparas

		Primiparas *n*=43403			Multiparas *n*=51648		
		Spontaneous (*n*=31 726, 73.1% of primiparas)	Induced (*n*=11 677, 26.9% of primiparas)	p-value*	Spontaneous (*n*=40 932, 79.3% of multiparas)	Induced (*n*=10 716, 20.7% of multiparas)	p-value*
Mode of delivery	Mode of delivery			<0.001			<0.001
	Vaginal	70.7%	54.7%		91.0%	83.7%	
	Instrumental	18.3%	18.9%		4.3%	6.3%	
	Urgent CS	10.0%	25.1%		4.3%	9.0%	
	Emergency CS	0.9%	1.3%		0.4%	1.0%	
Maternal characteristics	Maternal age ≥30	48.3%	54.7%	<0.001	70.8%	71.6%	0.099
	BMI before pregnancy ≥30	6.6%	14.4%	<0.001	9.7%	21.0%	<0.001
	Gestational age ≥42 weeks	2.8%	23.6%	<0.001	1.6%	15.3%	<0.001
	Prior caesarean section				13.2%	22.5%	<0.001
Delivery characteristics	Partner support	79.1%	77.7%	0.002	91.9%	90.6%	<0.001
	Use of epidural anesthesia	75.4%	75.6%	0.673	57.4%	70.3%	<0.001
	Prolonged labour	11.7%	18.4%	<0.001	4.1%	7.1%	<0.001
	Birth weight			<0.001			<0.001
	<3400g	44.8%	40.1%		31.2%	29.0%	
	3400-3799 g	34.1%	31.2%		35.4%	30.4%	
	3800-4199g	16.2%	20.6%		23.9%	26.7%	
	≥4200g	4.9%	8.1%		9.5%	13.9%	

*Chi-Square test used to detect significant differences between the distributions of spontaneous and induced labors

The models of the first phase are constructed using the univariate linear model in three phases separately for primiparous and multiparous women. In model 1 Table 2 the childbirth experience was explained through the main effect of the factor variables, labour induction and mode of birth. The confounding effects are controlled in model 2 adding the background characteristics – maternal age, BMI (Body Mass Index) before pregnancy, cohabitation as a proxy of partner support, gestational age, epidural anesthetic use, prolonged labour, and birth weight – in the model. An additional variable for multiparas was a prior caesarean section which received special attention in previous research [23]. Model 3 involves the interaction terms of labour induction and delivery modes. The coefficient estimates of these distinct models are then compared to comprehend the associations between the childbirth experience and induction in each of the mode of delivery groups. Spontaneous labour resulting in vaginal delivery was used as a reference group in the models. Coefficients in the models indicate the effect of each variable on the childbirth experience when compared to the reference group.

**Table 2:** The model coefficients and *p*-values for both primiparas and multiparas

		Primiparas						Multiparas					
		Model1		Model2		Model3		Model1		Model2		Model3	
	Parameter	B	Sig.	B	Sig.	B	Sig.	B	Sig.	B	Sig.	B	Sig.
	Intercept	8.26	0.000	8.78	0.000	8.77	0.000	8.72	0.000	8.88	0.000	8.88	0.000
Onset of labour	Induction	-0.34	0.000	-0.28	0.000	-0.20	0.000	-0.25	0.000	-0.22	0.000	-0.19	0.000
	Spontaneous^a^	0*		0*		0*		0*		0*		0*	
Mode of delivery	Emergency CS	-1.32	0.000	-1.52	0.000	-1.50	0.000	-1.88	0.000	-1.90	0.000	-1.70	0.000
	Urgent CS	-0.97	0.000	-0.93	0.000	-0.80	0.000	-1.03	0.000	-0.85	0.000	-0.73	0.000
	Instrumental delivery	-0.75	0.000	-0.57	0.000	-0.54	0.000	-0.91	0.000	-0.74	0.000	-0.74	0.000
	Vaginal delivery^a^	0*		0*		0*		0*		0*		0*	
Interaction terms	Induction * Emergency CS					-0.11	0.528					-0.51	0.004
	Induction*Urgent CS					-0.32	0.000					-0.34	0.000
	Induction* Intrumental delivery					-0.12	0.016					0.01	0.875
Confounding variables	Age of parturient ≥30 years			-0.10	0.000	-0.10	0.000			-0.03	0.021	-0.03	0.018
	BMI before pregnancy ≥30			-0.03	0.324	-0.03	0.353			-0.03	0.072	-0.03	0.082
	Partner support			0.09	0.000	0.09	0.000			0.02	0.475	0.01	0.510
	Gestational age			-0.04	0.216	-0.03	0.312			0.04	0.142	0.05	0.113
	Use of epidural anesthesia			-0.35	0.000	-0.35	0.000			-0.06	0.000	-0.06	0.000
	Prolonged labour			-0.37	0.000	-0.37	0.000			-0.47	0.000	-0.46	0.000
	Fear of childbirth			-0.41	0.000	-0.42	0.000			-0.22	0.000	-0.22	0.000
	Birth weight (categories)			-0.08	0.000	-0.08	0.000			-0.03	0.000	-0.03	0.000
	Prior CS									-0.06	0.001	-0.06	0.001

^a^ Reference group

Second, we analysed the risk of low VAS (≤5) between spontaneous and induced labours by each mode of delivery. The childbirth experience has been shown to cause undesirable consequences with low values of VAS. Thus, we pursued to scrutinise the risks of induced and spontaneous labour groups for ending up in this poor experience group. Furthermore, odds ratio (OR) estimates with 95% confidence intervals were calculated and compared between spontaneous and induced labour groups.

Third, we calculated relative risks with 95% confidence intervals to assess the prevalence of each mode of delivery between spontaneous and induced labours. According to the previous studies, each mode of delivery has its specific risks that should be accounted for when the induction trial comes to a decision. The risks of labour induction are emphasised particularly among multiparas with irregular an obstetric history. Taken together, we first assessed the effects of labour induction and mode of delivery on the childbirth experience. Second, we analysed the risk of low VAS between spontaneous and induced labours by each mode of delivery. And finally, we calculated the prevalence of each mode of delivery between spontaneous and induced labours.

### Missing values

The register data was complete except that the BMI before pregnancy values were lacking from 1 162 (2.7%) primiparas and 1 964 (3.8%) multiparas. According to a t-test, the mean of VAS of those lacking prepregnancy BMI did not significantly differ from the mean of VAS of complete data.

### Patient and public involvement

In our register-based data patient and public involvement was not feasible. The data used in this study was collected retrospectively from the registers of Helsinki University Hospital and the Finnish Institute of Health and Welfare. The Institutional review board gave the permission (HUS/483/2020) to use the data and waived the requirement of the informed consent and a separate Ethical Committee review for the study since it was a purely register-based study. Therefore, the informed consents of registered patients were not required for this study.

## Results

The inclusion criteria for this study are depicted in Fig. 1. We included all hospital childbirths of term (≥37 weeks) singleton pregnancy with live infant. Childbirth experience and labour induction being of interest, we excluded the childbirths of elective caesarean sections and those lacking VAS. The final data consisted of 95 051 parturients including 43 403 primiparas and 51 648 multiparas. The share of induced labours in primiparas was 27% and in multiparas 21%.

The characteristics of spontaneous and induced labour groups are presented in Table 1 in categories used in covariance analysis. Difference between distributions of induced and spontaneous groups was detected in all variables except the use of epidural anesthesia among primiparas and maternal age among multiparas. The maternal factors such as mean age (30.1 vs. 29.1 years for primiparas, *p*<0.001; 32.3 vs. 32.0 years for multiparas, *p*<0.001) and mean BMI before pregnancy (24.7 vs. 23.2 for primiparas, *p*<0.001; 26.0 vs. 23.9 for multiparas, *p*<0.001) were higher in the induced labour group compared to the spontaneous onset of labour group. Gestational age, being one of the common indications for labour induction, was higher in the induced labour group (40.2 vs. 39.7 for primiparas, *p*<0.001; 39.8 vs. 39.7 for multiparas, *p*<0.001). Prolonged labour was diagnosed more frequently with induction than with spontaneous labour (*p*<0.001). The average birth weight was higher for the labour induction group for both primiparas (3516g vs. 3465g, *p*<0.001) and multiparas (3661g vs. 3616g, *p*<0.001). In our data preeclampsia (ICD10 codes O11, O14, and O15.0) was diagnosed in 1000 (2.3%) of primiparous and in 431 (0.8%) of multiparous women. Gestational diabetes (ICD10 code O24.4) was diagnosed in 5926 (13.7%) primiparous and in 8415 (16.3%) multiparous women.

The Medical Birth Register does not include complete data about different techniques of induction used in hospitals. However, the used techniques of induction separately in primiparas and multiparas were artificial rupture of membranes (79% and 88%, of induced labours), prostaglandin (28% and 19%) and oxytocin (89% and 74%).

### Effects of labour induction and mode of delivery on the childbirth experience

#### Main effects

The coefficients of different regression covariance analysis models for primiparas and multiparas are presented in Table 2. For both parity groups, the effect of labour induction was negative (β_P_=-0.34, *p*<0.001; β_M_=-0.25, *p*<0.001) on the childbirth experience when the actual mode of delivery was included in model 1. For primiparas, the coefficient of emergency caesarean section (β=-1.32, *p*<0.001) was strongest, followed by urgent caesarean section (β=-0.97, *p*<0.001) and instrumental delivery (β=-0.75, *p*<0.001) when compared to the vaginal delivery group.

The coefficients in the model of multiparas were -1.88 (*p*<0.001), -1.03 (*p*<0.001), and -0.91 (*p*<0.001), for emergency and urgent caesarean sections and instrumental delivery, respectively. When confounding variables were adjusted in model 2, the negative effect of labour induction faded slightly compared to the basic model being -0.28 for primiparas and -0.22 for multiparas (model 1).

#### Control variables

Six statistically significant variables – maternal age, partner support, use of epidural anesthesia, prolonged labour, fear of childbirth and birth weight – were detected in the model 2 of primiparas. The strongest negative effect had the fear of childbirth (β=-0.41, *p*<0.001), prolonged labour (β=-0.37, *p*<0.001) and use of epidural anesthesia (β=-0.35, *p*<0.001). Significant control variables in the model for multiparas were equal compared to primiparas, except for partner support, which lost its positive effect among multiparas. Prolonged labour had the strongest negative effect (β=-0.47, *p*<0.001) on the childbirth experience followed by fear of childbirth (β=-0.22, *p*<0.001) in model 2 for multiparas, while the effects of other significant control variables were smaller.

#### Interaction effects

Two significant interaction effects were observed in model 3 for primiparas. The induced labour resulting in instrumental delivery had negative interaction effect (β=-0.12, *p*=0.016) on the childbirth experience as well as the urgent caesarean section (β=-0.32, *p*<0.001) when adjusted for confounding variables. The negative effect of emergency caesarean section was statistically insignificant, even though its coefficient was close to the effect of instrumental delivery. This finding could be affected by the low number of emergency caesarean sections in both groups. In multiparas, both urgent (β=-0.34, *p*<0.001) and emergency caesarean sections (β=-0.51, *p*=0.004) had negative interaction effects with induction on the childbirth experience.

**Fig. 2:**
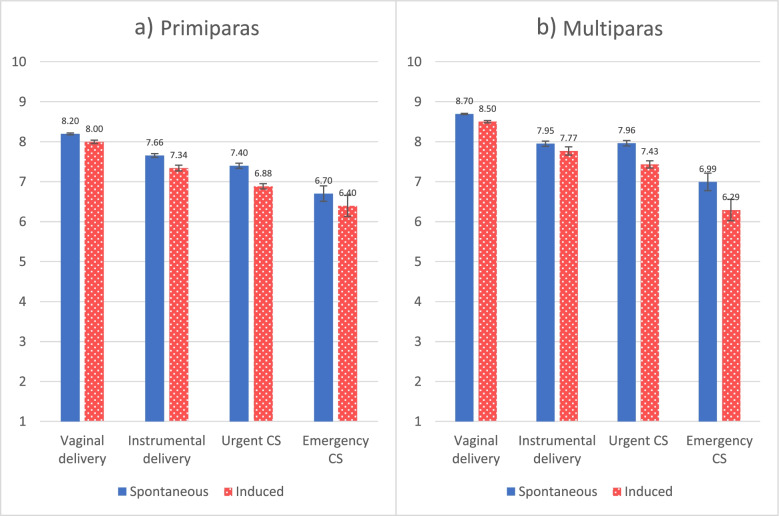
The adjusted means of VAS with 95% confidence intervals for spontaneous and induced labours in each mode of delivery (corresponding data in a numerical Table 6 is available in a supplementary file)

The adjusted means with 95% confidence intervals of the childbirth experience in each mode of delivery were calculated using model 3 for both parity groups (Fig. 2a and b). Following the coefficients shown in Table 2, the difference between spontaneous and induced labours was larger for instrumental delivery or urgent caesarean among primiparas (Fig. 2a). The overlapping confidence intervals of means revealed that VAS difference disappeared in the emergency caesarean section group.

Regarding multiparas, the mean differences between spontaneous and labour induction groups were considerably larger with urgent and emergency caesarean sections compared to both unassisted and instrumental vaginal delivery groups (Fig. 2b). The labour induction resulting to the unplanned caesarean section produced a lower childbirth experience than labour prolonged labour with spontaneous onset. These visual views coincided with the coefficients in Table 2.

#### Poor childbirth experience

Low VAS scores indicating poor childbirth experience (≤5) were presented for spontaneous and induced labour groups in each mode of delivery separately. The risk of low VAS was lowest for vaginal delivery with spontaneous onset of labour (5.3%) and highest for emergency caesarean section with induced onset of labour (26.5%) for primiparas and 2.4% and 35.3% for multiparas, respectively. Labour induction was associated with higher odds of perceiving a poor childbirth experience for each mode of delivery and both parity groups (Table 3). Comparing the risks of induced and spontaneous labours in mode of delivery groups for primiparas the OR varied from 1.54 to 1.70 except the estimate for emergency caesarean (OR=1.08 [0.69–1.68]). Corresponding estimates of multiparas varied from 1.43 to 1.77 in all modes of delivery except for emergency caesarean section (OR=1.70 [0.98–2.94]). These results imply that 10 poor experiences in spontaneous labour group equals at least 15 poor childbirth experiences in the induced labour group independent of the actual mode of delivery (excluding the emergency caesarean section).

**Table 3:** Childbirths with low VAS (≤5) in each mode of delivery for induced and spontaneous labour groups and corresponding odd ratio estimates with 95% confidence intervals

	Spontaneous labour		Induced labour			
**Primiparas (*n*=4252)**	**n**	**%**	**n**	**%**	**OR**	**95% CI**
Vaginal delivery (both)	1183	5.3	503	7.9	1.54	1.38 to 1.71
Instrumental delivery	774	13.3	444	20.1	1.64	1.44 to 1.87
Urgent CS	511	16.1	721	24.6	1.70	1.50 to 1.93
Emergency CS	75	25.0	41	26.5	1.08	0.69 to 1.68
**Multiparas (*n*=1993)**	**n**	**%**	**n**	**%**	**OR**	**95% CI**
Vaginal delivery (both)	895	2.4	369	4.1	1.74	1.54 to 1.97
Instrumental delivery	185	10.5	97	14.3	1.43	1.10 to 1.87
Urgent CS	198	11.2	176	18.2	1.77	1.42 to 2.21
Emergency CS	37	24.3	36	35.3	1.70	0.98 to 2.94

### Labour induction and risk to operative deliveries

The effect of mode of delivery on the childbirth experience was larger than the effect of labour induction. Labour induction was associated with increased risk for urgent (RR=2.51 with 95% CI [2.40, 2.62]) and emergency caesarean sections (RR=1.40 [1.16, 1.70]) while there was no association to the likelihood of instrumental delivery among primiparas (Table 4). Among multiparas, labour induction more than doubled the risk for both urgent (RR=2.08 [1.93, 2.25]) and emergency caesarean sections (RR=2.56 [2.00 3.29]), while the risk of instrumental delivery (RR=1.46 [1.34, 1.59]) was increased by nearly 50%. Correspondingly, labour induction was associated with a lower likelihood to achieve vaginal delivery, while the risk ratios are below one for both primiparas (RR=0.77 [0.76, 0.79]) and multiparas (RR=0.92 [0.91, 0.93]).

**Table 4:** The percentage distributions of actual mode of deliveries following spontaneous and induced labours. The corresponding relative risk with 95 % confidence interval for belonging to each group when labour was induced

	Spontaneous labour		Induced labour			
**Primiparas (*n*=43 403)**	**n**	**%**	**n**	**%**	**RR**	**95% CI**
Vaginal delivery (both)	22 428	70.7	6 382	54.7	0.77	0.76 to 0.79
Instrumental delivery	5 819	18.3	2 206	18.9	1.03	0.99 to 1.08
Urgent CS	6 179	10.0	2 934	25.1	2.51	2.40 to 2.62
Emergency CS	300	0.9	155	1.3	1.40	1.16 to 1.70
**Multiparas (*n*=51 648)**	**n**	**%**	**n**	**%**	**RR**	**95% CI**
Vaginal delivery (both)	37 239	91.0	8 971	83.7	0.92	0.91 to 0.93
Instrumental delivery	1 767	4.3	676	6.3	1.46	1.34 to 1.59
Urgent CS	1 774	4.3	967	9.0	2.08	1.93 to 2.25
Emergency CS	152	0.4	102	1.0	2.56	2.00 to 3.29

## Discussion

Our study revealed three main findings: 1) The induction of labour in general impaired the childbirth experience, excluding primiparas who underwent an emergency caesarean section. Poor childbirth experience risk is higher in the induced labour group for both primiparas and multiparas. 2) Operational deliveries were perceived more negative if preceded by labour induction, as one fifth of induced primiparas and one seventh of induced multiparas valued their experience as poor. 3) We found a decreased likelihood to achieve vaginal delivery among women who had induced labour.

To compare previous studies assessing childbirth experience, we gathered their main results in Table 5. In our study, we show that induction of labour produces impaired childbirth experience, which is in line with a previous study [15]. The risk of achieving a poor experience was analysed in several studies [2, 7, 8, 24]. Inconsistencies in measurement of experience and definition of a poor or negative experience exist between the studies. However, the overall findings in the previous studies are in line with ours.

**Table 5:** The effects of induction and mode of delivery to childbirth experience in previous studies

	Measure	Exposure	Association
**Induction**			
Adler et al. (2020) [8]	VAS (<5 vs. ≥5)	Induction (ref. spontaneous)	aOR = 1.6 (1.4-1.9)
Falk et al. (2019) [7]	VAS (1-3 vs. 4-10)	Induction (ref. spontaneous)	aOR = 1.69 [1.44-1.98]
Hildingsson et al. (2011) [24]	Negative (vs. positive) birth experience	Induction (ref. spontaneous)	aOR = 1.5 [1.0-2.3]
Schaal et al. (2019) [15]	CEQ (Overall Score)	Induction	m = 3.00 (SD=0.30),
		Spontaneous	m = 3.10 (SD=0.29), *p*=0.023
Shetty et al. (2005)	Satisfied with labour (4-5 vs. 1-3)	Induction (ref. spontaneous)	RR = 0.89 (0.80-0.96)
Waldenström et al. (2004) [2]	Negative (1-2) birth experience (vs. positive, 3-7)	Induction (ref. spontaneous)	RR = 2.4 [1.7-3.4]
**Mode of delivery**			
Adler et al. (2020) [8]	VAS (<5 vs. ≥5)	CS (ref. SVD)	aOR = 4.5 (3.7–5.5)
		IVD (ref. SVD)	aOR = 3.3 (2.7–4.0)
Blomquist et al. (2011) [17]	VAS (0-100); Salmon score: Fulfillment/ Distress/ Difficulty	Planned CS	90.9; 0.23/-0.24/-0.54
		Unplanned CS	73.9; -0.61/0.62/0.41/
		SVD	86.8; 0.14/-0.19/0.03
		OVD	76.2; 0.11/0.05/0.20
Bossano et al. (2017) [16]	Salmon score: Fulfillment/ Distress/ Difficulty	Planned CS	0.53/-0.16/-0.43
		Unplanned CS	0.02/0.20/0.17
		SVD	0.47/-0.52/-0.13
		OVD	0.15/0.02/0.47
Carquillat et al. (2016) [18]	NRS from 0 (=very bad experience) to 10 (=very good experience)	SVD	7.94 (2.14)
		IVD	6.96 (2.43)
		Elective CS	7.00 (1.97)
		Emergency CS	5.12 (3.18)
Falk et al. (2019) [7]	VAS(1-3)	IVD (ref. SVD)	aOR = 2.9 (2.3–3.6)
		Emergency CS (ref. SVD)	aOR = 4.0 (3.3–4.9)
Hildingsson et al. (2011) [24]	Negative vs. positive birth experience	IVD (ref. SVD)	OR = 1.4 (0.7–2.4)
		Unplanned CS (ref. SVD)	OR = 3.1 (1.9–5.0)
Kempe & Vikström-Bolin (2020) [19]	Average VAS (0-10)	SVD	8.18
		OVD	6.85
		Unplanned CS	7.12
Rijnders et al. (2008)	Negative recall	OVD or Unplanned CS (ref. SVD)	aOR = 2.6 (1.6–4.1)
Spaich et al. (2013) [25]	Good/very good' satisfaction with childbirth	Normal delivery	89 %
		Primary CS	94 %
		Secondary CS	87 %
		Emergency CS	89 %
		OVD	93 %
Waldenström et al. (2004) [2]	Negative vs. positive birth experience (7-point scale 1-2 vs. 3-7)	IVD (ref. SVD)	RR = 4.0 (2.7–5.9)
		Elective CS (ref. SVD)	RR = 1.1 (0.5–2.4)
		Emergency CS (ref. SVD)	RR = 5.7 (4.1–7.9)

*CEQ* Childbirth Experience Questionnaire, *SVD* spontaneous vaginal delivery, normal vaginal, *IVD* instrumental vaginal delivery, *OVD* operative vaginal delivery, *CS*caesarean section, *NRS* numeric rating scale

In previous studies, the role of the mode of delivery is mostly parallel to our findings. Two studies comparing average experience in mode of delivery groups [17, 18] confirm our finding that vaginal delivery produces the most positive and unplanned caesarean the most negative experience when elective caesarean is not considered. One study did not find association between the modes of delivery [25] and other findings were inconsistent with ours [19], which may be due to the sampling inadequacy (there were only 34 observations in CS group). The risk ratios of negative experience in previous studies indicate similar associations between the mode of delivery and childbirth experience regardless of the fact that we did not compute equivalent risk ratios for modes of delivery in our study.

In our study, we found that induced labour resulting in vaginal delivery was associated with a more positive childbirth experience on average than operational deliveries with spontaneous onset of labour. This leads to the conclusion that successful induction resulting in vaginal delivery does not ruin the positive childbirth experience. However, induction increases the risk for operational delivery which is also shown in previous studies [11, 12, 26, 27]. Operational delivery is likely to impair the childbirth experience, as supported by our findings that the childbirth is experienced more negatively if the labour induction results in caesarean section. It is possible that parturient feel frustration when failing to give vaginal birth together with discomfort and pain due to the unsuccessful labour induction. Our novel approach to evaluate the association of childbirth experience and induction in the mode of delivery should be explored more thoroughly to confirm the findings.

Our study is based on a large comprehensive data set of 95 051 childbirths and it covers 90% of eligible parturients in the study population. The data population covers nearly one third of all Finnish childbirths during the years 2012–2018. The VAS collection was an established routine practice of care, and its simplicity led to minimising the selection bias. The simplicity of the measure also attenuates the possible challenges due to the lack of shared language between the caregiver and the parturient. The national comprehensive Medical Birth Register in combination with the maternal childbirth experience provides reliable data to analyse the associations between the maternal childbirth experience and various factors behind it [28]. Despite the distinct levels of primiparas and multiparas, the resulting nearly identical patterns of childbirth experience confirm the reliability of our data.

Our study also has limitations. Our main outcome measure VAS does not incorporate the multidimensionality of the childbirth experience. However, VAS has been used to measure childbirth experience and satisfaction with childbirth in several previous studies [5–8, 19]. Furthermore, VAS is moderately correlated with the highly established and validated W-DEQ (Wijma Delivery Expectancy/Experience Questionnaire) scale for measuring childbirth experience [5, 29].

The maternal childbirth experience was collected before the patient was discharged from the post-partum care unit, usually less than 72 hours after the delivery. This could have had an effect on the results, since traumatic experience might take longer to process in the mind [30]. Maimburg and colleagues [31] found similar tendency that women told more negative experiences five years after delivery although women changing their mind had more often experienced operative delivery. In our study setting this would lead to even greater difference. However, there are results that indicate the consistency of childbirth experience from a few days postpartum to few months afterwards [6, 17]. We did not account potential effects of preeclampsia or gestational diabetes which may cause some bias to the results. It has been shown that although gestational diabetes seems to increase the risk of induction, it has no effect on childbirth experience [8]. In the absence of research focusing on the relation between preeclampsia and childbirth experience, the study comparing the mental health indicators after normotensive and hypertensive pregnancies suggests that preeclampsia rises the risk of traumatic childbirth [32].

While the trial of induction is mostly based on obstetrical arguments (suspected fetal macrosomia, post-term gestation, or fear of childbirth) the results of this study are complicated by these underlying factors. By using detailed register data, we attempted to control these effects on the childbirth experience.

## Conclusion

Labour induction produces a more negative childbirth experience for both primiparas and multiparas. Women who had induced labour had also decreased likelihood to achieve vaginal delivery. Although the negative effect of labour induction is detected for all modes of delivery, the highest risk for a poor experience is when labour induction results in an operative delivery. Our findings can be used in decision-making related to parturients requesting labour induction without clear obstetrical indications. Furthermore, the parturients facing cumulative obstetrics interventions require special support and counselling from obstetric care during and after delivery.

## Supplementary Information

**Additional file 1.**

## Data Availability

The data that support the findings of this study are available from Finnish Institution Health and Welfare and Helsinki University Hospital but restrictions apply to the availability of these data, which were used under license for the current study, and so are not publicly available.

## Abbreviations

VAS: Visual Analogue Scale
OR: Odds ratio
RR: Risk ratio
BMI: Body mass index
SVD: Spontaneous vaginal delivery
IVD: Instrumental vaginal delivery
OVD: Operative delivery
CS: Caesarean section
UCS: Urgent caesarean section
ECS: Emergency caesarean section
CEQ: Childbirth Experience Questionnaire
NRS: Numeric rating scale
W-DEQ: Wijma Delivery Expectancy/Experience Questionnaire
