# Supplementary material for: Maternal childbirth experience and induction of labour in each mode of delivery: a retrospective seven-year cohort study of 95,051 parturients in Finland
Source: BMC Pregnancy Childbirth. 2022 Jun 23;22:508. doi: 10.1186/s12884-022-04830-9 (PMC9229460; doi:10.1186/s12884-022-04830-9)
Supplement: Supplementary file 1 — Additional file 1. [file 12884_2022_4830_MOESM1_ESM.docx]

## SUPPLEMENT

Table 6. The adjusted means of VAS with 95% confidence intervals for spontaneous and induced labours in each mode of delivery (data is presented in figures 2a and 2b).

|  | Primiparas (n=42 226) | | | | Multiparas (n=49 672) | | | |
| --- | --- | --- | --- | --- | --- | --- | --- | --- |
|  | Spontaneous (n=30 814) | | Induced (n=11 412) | | Spontaneous (n=39 296) | | Induced (n=10 376) | |
|  | Mean | 95% CI | Mean | 95% CI | Mean | 95% CI | Mean | 95% CI |
| Vaginal delivery | 8.196 | 8.173 to 8.219 | 7.997 | 7.955 to 8.039 | 8.695 | 8.680 to 8.709 | 8.501 | 8.473 to 8.530 |
| Instrumental delivery | 7.656 | 7.611 to 7.701 | 7.342 | 7.269 to 7.413 | 7.951 | 7.885 to 8.017 | 7.768 | 7.664 to 7.871 |
| Urgent CS | 7.398 | 7.335 to 7.463 | 6.884 | 6.818 to 6.950 | 7.962 | 7.894 to 8.030 | 7.432 | 7.343 to 7.522 |
| Emergency caesarean | 6.702 | 6.508 to 6.896 | 6.395 | 6.131 to 6.659 | 6.993 | 6.775 to 7.213 | 6.291 | 6.025 to 6.558 |
